# DEVELOPMENT OF THE MEDICARE BENEFICIARY CARE SETTING TRAJECTORY DATABASE

**DOI:** 10.1093/geroni/igad104.1463

**Published:** 2023-12-21

**Authors:** Olga Jarrín, Anum Zafar, Hyosin Kim, Haiqun Lin

**Affiliations:** Rutgers, The State University of New Jersey, New Brunswick, New Jersey, United States; Rutgers, The State University of New Jersey, New Brunswick, New Jersey, United States; Oregon State University, Corvallis, Oregon, United States; Rutgers, The State University of New Jersey, Newark, New Jersey, United States

## Abstract

Despite national efforts to shift from fragmented to value-based/coordinated care, there is a lack of understanding of older adults’ longitudinal aging experience across diverse healthcare settings in their last years. To improve patient care and gain insights into areas for improvement in care coordination and management, it is crucial to have evidence about older adults’ longitudinal care experience and healthcare transitions. This study outlines the development of the Care Setting Trajectory File, which combines information on service dates and discharge destinations from various Medicare administrative, assessment, and claims data. The data sources include MedPAR, Hospice, Minimum Data Set (MDS), MDS-Swing Bed (SB), Outcome and Assessment Information Set (OASIS), and the Inpatient Rehabilitation Facility Patient Assessment Instrument (IRF-PAI). The Care Setting Trajectory File contains a day-level summary of care settings, including inpatient hospice, inpatient-short stay, inpatient-long stay, inpatient-SNF, inpatient-VA, inpatient, rehab facility, nursing home, home health, hospice home, and home. This day-level file can then be utilized at various levels of granularity, i.e., monthly, quarterly, or yearly periods, for further analysis. For illustration, the file construction method is detailed for a cohort of Medicare decedents who died in 2019. Examples of how this analytic file can be used to generate important insights for policymakers and healthcare providers on improving care coordination, continuity, and quality of life for older adults with serious illnesses will be discussed.

